# 2562. Retrospective Analysis of Cefepime Intravenous Push versus Extended Infusion for the Treatment of Neutropenic Fever

**DOI:** 10.1093/ofid/ofad500.2179

**Published:** 2023-11-27

**Authors:** Thomas J Rust, Tracy M Krause, Stephen Saw

**Affiliations:** Hospital of the University of Pennsylvania, Philadelphia, Pennsylvania; Hospital of the University of Pennsylvania, Philadelphia, Pennsylvania; Hospital of the University of Pennsylvania, Philadelphia, Pennsylvania

## Abstract

**Background:**

The outcomes associated with intravenous push (IVP) cefepime compared to extended infusion (EI) have not yet been studied. The objective of this study was to evaluate the effectiveness of IVP versus EI cefepime in patients with neutropenic fever.

**Methods:**

This was a retrospective, single-center, cohort study evaluating hematopoietic stem cell transplant (HSCT) recipients who received IVP or EI cefepime for at least 48 hours for the treatment of neutropenic fever. Patients were excluded if they were admitted to an intensive care unit (ICU) at the time of cefepime initiation, required renal replacement therapy, received an IVP dose < 2 grams, or grew a cefepime-resistant organism from initial cultures. Patients were grouped 2:1 EI-to-IVP according to weight >100 kg and autologous or allogeneic HSCT. The primary outcome was defervescence at 72 hours. Secondary outcomes included 30-day in-hospital mortality, ICU transfer within 72 hours, ICU length of stay (LOS), hospital LOS, breakthrough infection, and escalation of gram-negative therapy within 72 hours. Data was analyzed using independent t-test or Wilcoxon Rank Sum test if continuous and chi-square test or Fisher’s Exact test if nominal.

**Results:**

Of the 120 patients meeting eligibility criteria, 40 patients received IVP cefepime and 80 patients received EI cefepime. All baseline demographics were balanced between groups with the exception of time from cefepime order entry to first dose administration (Table 1). Concomitant medication administrations were also similar between groups apart from antipyretic administration (Table 2). Defervescence within 72 hours occurred in 28 (70%) patients treated with IVP and 51 (63.8%) patients treated with EI (*p* = 0.496). No patients experienced in-hospital mortality and median hospital LOS was not significantly impacted by IVP vs EI administration (18 vs 17.5 days, *p* = 0.158). None of the additional secondary outcomes studied were significantly different between groups (Table 3).

**Figure:**
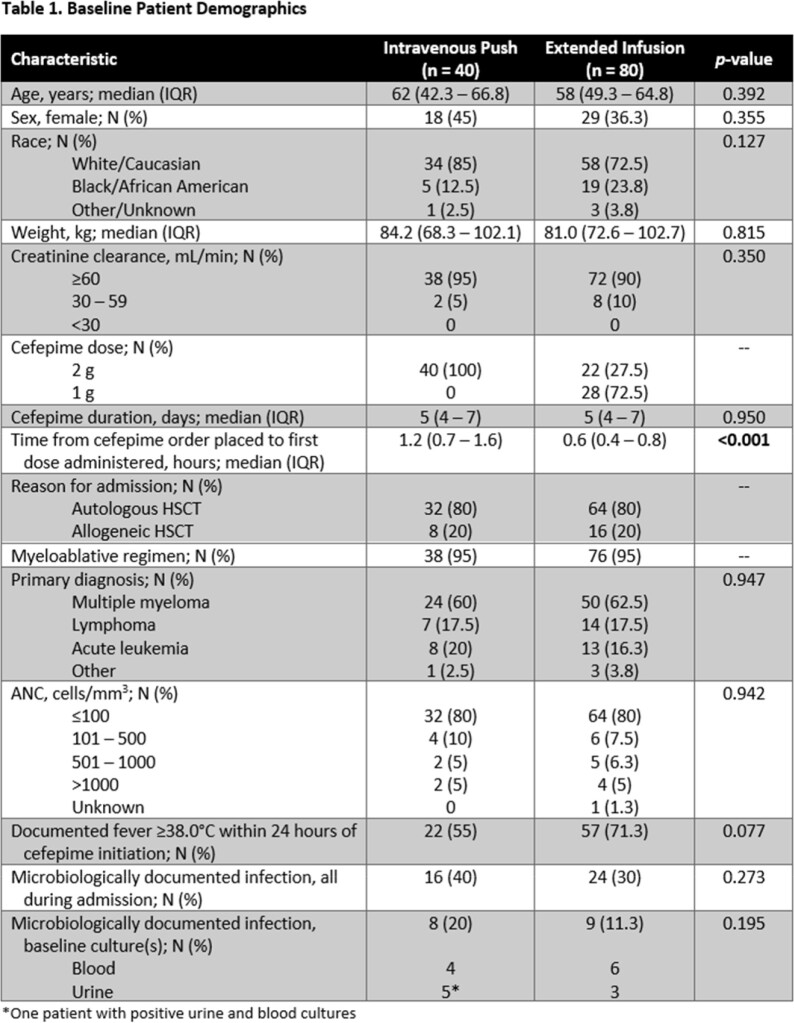

**Figure:**
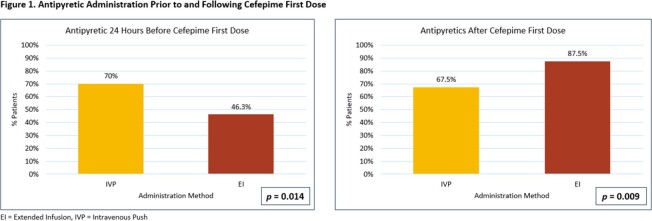

**Figure:**
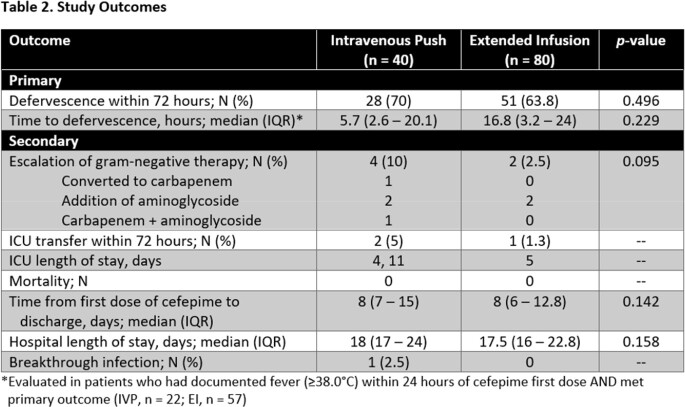

**Conclusion:**

The current study showed no significant differences in clinical outcomes when administering cefepime as IVP or EI for the treatment of neutropenic fever. Larger studies and/or randomized controlled trials are necessary to confirm the findings of this study.

**Disclosures:**

**All Authors**: No reported disclosures

